# Bundibugyo Virus Disease Outbreak and Cross-Border Response in the Democratic Republic of the Congo and Uganda, May-June 2026

**DOI:** 10.7759/cureus.112459

**Published:** 2026-07-11

**Authors:** Prosper Akankwasa, Jackson Kakooza, Wamala N Kisaakye, Faith Nabushawo, Shakirah Mutonyi, Catherine Lewis

**Affiliations:** 1 Department of Obstetrics and Gynecology, Kampala International University Western Campus, Ishaka Bushenyi, UGA; 2 Department of Surgery, Kampala International University Western Campus, Ishaka Bushenyi, UGA; 3 Department of Public Health, Infectious Diseases Institute, Kampala, UGA; 4 Department of Internal Medicine, Old Kampala Hospital, Kampala, UGA; 5 Department of Surgery, St. Joseph's Hospital Kitovu, Masaka, UGA

**Keywords:** bundibugyo virus disease, democratic republic of the congo, ebola, outbreak, uganda

## Abstract

Bundibugyo virus disease (BVD) is a high-consequence filovirus infection with no approved vaccine or specific therapeutic, making early detection, supportive care, infection prevention, and coordinated public health action essential. This editorial is a rapid narrative synthesis of publicly available outbreak information from the WHO Disease Outbreak News, the Centers for Disease Control and Prevention (CDC) reports, the Uganda Ministry of Health updates, and the WHO African Region situation materials. We prioritized official reports because the event was rapidly evolving and because case counts, contact-tracing indicators, and health-zone classifications were being updated as surveillance and laboratory capacity expanded. This editorial summarizes the May-July 2026 BVD outbreak involving the Democratic Republic of the Congo (DRC) and Uganda and highlights public health lessons for cross-border preparedness. We reviewed official WHO, CDC, Uganda Ministry of Health, and WHO African Region reports, with data reviewed through July 3, 2026. WHO was alerted on May 5, 2026, to a high-mortality illness cluster in Mongbwalu Health Zone, Ituri Province, DRC. The DRC and Uganda declared outbreaks on May 15, 2026, and the WHO determined that the event constituted a public health emergency of international concern. Response measures included surveillance, laboratory expansion, isolation and supportive care, contact tracing, infection prevention and control, safe burial, community engagement, cross-border coordination, and preparation for ethically approved trials of candidate countermeasures. This outbreak shows that border-linked surveillance must be integrated with first-contact facility readiness, health-worker protection, trusted community engagement, continuity of essential services, complete contact follow-up, and trial-ready systems rather than reliance on generic Ebola plans or indiscriminate travel restrictions.

## Editorial

Investigation and results

On May 5, 2026, the WHO received reports of an unusual cluster of severe illness and deaths, including deaths among health workers, in Mongbwalu Health Zone, Ituri Province, Democratic Republic of the Congo (DRC). On May 14, the Institut National de Recherche Biomédicale tested 13 blood specimens submitted from Rwampara Health Zone; eight were positive for Bundibugyo virus on May 15. The DRC and Uganda declared outbreaks on May 15, and the WHO determined on May 17 that the event constituted a public health emergency of international concern [1,2].

Initial transmission was concentrated in Ituri Province, particularly Bunia, Rwampara, Mongbwalu, and Nyankunde health zones, and later expanded into North Kivu and South Kivu. The situation continued to evolve rapidly. By July 1, 2026, the DRC had reported 1,460 confirmed cases and 452 deaths from 36 health zones across Ituri, North Kivu, and South Kivu provinces. Ituri remained the most affected province, accounting for 1,333 (91.3%) of confirmed cases and 380 (84.0%) of deaths. Among the confirmed cases, 102 were reported among healthcare workers. Authorities had identified 10,821 contacts in Ituri and North Kivu, with follow-up rates of 83.2% and 81.0%, respectively. Insecurity, population displacement, delayed access to affected communities, health-facility incidents, and expanding surveillance and testing capacity complicated the interpretation of apparent epidemic growth [3].

Uganda’s first confirmed infections were associated with travel from the DRC. By July 2, 2026, Uganda had reported 20 confirmed cases, including two deaths and one probable fatal case. Fifteen confirmed cases were imported, and five reflected secondary transmission among contacts and health workers linked to imported cases from the DRC. Cases occurred in Kampala and Wakiso districts; four health workers were affected. No community transmission had been documented, and no new confirmed case had been reported since June 21. Of 831 contacts listed as of June 28, 821 had completed 21 days of follow-up by July 2 [3].

The investigation identified two linked operational risks. First, infected travelers and care seekers crossed national and district boundaries before or during illness, including through border, trade, transport, referral, and informal movement networks. Second, several exposures occurred in healthcare settings, where nonspecific early symptoms can delay recognition and where procedures involving bodily fluids increase risk. The international spread of risk was further illustrated by a laboratory-confirmed case of Bundibugyo virus disease (BVD) reported in France on June 24, 2026, in a physician returning from the DRC. These findings were consistent with previous Ugandan preparedness assessments that identified numerous formal and informal crossings and variable infection-prevention readiness in high-risk facilities [3-6].

Public health response

The DRC and Uganda, with support from the WHO, the Africa Centres for Disease Control and Prevention (Africa CDC), the United States Centers for Disease Control and Prevention (US CDC), and partner organizations, expanded surveillance, laboratory testing, isolation and optimized supportive care, contact tracing, safe and dignified burial, risk communication, and infection prevention and control. Uganda intensified facility and community case finding in Kampala and Wakiso and strengthened screening and alert management at points of entry, along transport corridors, and in health facilities likely to receive febrile travelers or referred patients [3,4,7].

On May 22-23, health ministers and technical partners from the DRC, Uganda, and South Sudan agreed to coordinate cross-border surveillance, laboratory referral, contact-tracing information, case management, infection prevention and control, and risk communication. WHO advised against general travel or trade restrictions and emphasized rapid information sharing, targeted border-health measures, and sustained surveillance rather than border closure [2,3,8].

No vaccine or specific therapeutic is approved for BVD. Early optimized supportive care, therefore, remains central. WHO expert groups recommended that candidate vaccines, monoclonal antibodies, and antivirals be evaluated only within ethically approved clinical trials. Trial readiness requires preapproved protocols, regulatory and ethics pathways, trained sites, pharmacovigilance, secure supply chains, transparent community engagement, and capacity to protect essential services while research is implemented during an emergency [3,9,10].

Discussion

This outbreak demonstrates how a filovirus event can spread through a combined border, transport, referral, and healthcare network. The Ugandan pattern of predominantly imported infections with limited secondary transmission underscores the value of rapid case detection and contact follow-up, but health worker infections indicate that triage and infection prevention must operate across all first-contact facilities, not only designated Ebola treatment units. The editorial value of this outbreak is therefore not only the case count but the operational lesson that preparedness fails when border surveillance, facility triage, laboratory referral, occupational safety, and community reporting are planned as separate systems [3-5,7].

Preparedness at the DRC-Uganda interface should connect community alerts, formal and informal crossing points, ambulance and referral systems, private clinics, laboratories, and referral hospitals. Screening alone will miss people who use informal routes or develop symptoms after arrival. Facilities should maintain travel and exposure assessment, immediate separation of suspected cases, appropriate personal protective equipment, safe specimen handling, rapid notification, and pre-identified referral pathways while preserving emergencies, maternity, childhood illness care, and other essential services. Maintaining essential services is not secondary to outbreak control; it reduces fear, preserves trust, and prevents avoidable morbidity from non-Ebola conditions [5,6].

Health-worker protection should be treated as a core outbreak-control indicator. Recurrent exposures among health workers suggest the need for routine fever-and-travel triage, refresher training for private and public facilities, supervision of donning and doffing, waste-management readiness, occupational exposure reporting, psychosocial support, and reliable supplies of personal protective equipment. These measures are especially important where malaria, typhoid, pregnancy-related complications, and other common conditions can initially resemble early BVD and delay isolation.

Community trust is equally operational. Contact tracing and safe burial depend on timely reporting and cooperation; coercive or poorly explained measures can drive concealment and resistance. Response teams should use local languages and work with community health workers, religious and traditional leaders, women’s groups, transport associations, market leaders, miners, and survivors. Exposure data should be disaggregated by sex, occupation, pregnancy status, caregiving role, and health-worker category so that prevention addresses who performs high-contact care rather than assuming risk from sex alone [11].

The outbreak also provides a policy lesson for medical countermeasures. Because BVD lacks an approved vaccine or disease-specific therapeutic, emergency research should be prepared before products arrive: trial protocols, ethics approvals, regulatory pathways, data systems, safety monitoring, cold-chain and drug logistics, community consent processes, and survivor follow-up should be pre-positioned in high-risk settings. Otherwise, promising candidates may arrive too late or be used outside a framework capable of generating reliable evidence [9,10].

Limitations

This editorial relies on official public health reports and situation updates rather than primary surveillance records. The interpretation is therefore limited by reporting delays, changing case definitions, laboratory testing backlogs, incomplete contact follow-up, insecurity-related access constraints, and reclassification of cases, contacts, and health zones as investigations mature. Because the outbreak was still evolving after the manuscript’s data cutoff, the figures reported here should be read as a time-bounded summary of information reviewed through July 3, 2026, not as final outbreak totals.

Conclusion

The immediate public health priorities are sustained cross-border surveillance, complete contact follow-up, decentralized laboratory access, reliable triage and infection prevention in all first-contact health facilities, safe and dignified burial, community-led communication, health-worker protection, continuity of essential services, and trial-ready systems for Bundibugyo-specific countermeasures. These measures are more actionable than relying on generic Ebola plans or indiscriminate travel restrictions, and they should be embedded in routine preparedness across the DRC-Uganda border corridor before the next cross-border alert occurs.
